# Selection on Plant Male Function Genes Identifies Candidates for Reproductive Isolation of Yellow Monkeyflowers

**DOI:** 10.1371/journal.pgen.1003965

**Published:** 2013-12-05

**Authors:** Jan E. Aagaard, Renee D. George, Lila Fishman, Michael J. MacCoss, Willie J. Swanson

**Affiliations:** 1 Department of Genome Sciences, University of Washington, Seattle, Washington, United States of America; 2 Division of Biological Sciences, University of Montana, Missoula, Montana, United States of America; Harvard University, United States of America

## Abstract

Understanding the genetic basis of reproductive isolation promises insight into speciation and the origins of biological diversity. While progress has been made in identifying genes underlying barriers to reproduction that function after fertilization (post-zygotic isolation), we know much less about earlier acting pre-zygotic barriers. Of particular interest are barriers involved in mating and fertilization that can evolve extremely rapidly under sexual selection, suggesting they may play a prominent role in the initial stages of reproductive isolation. A significant challenge to the field of speciation genetics is developing new approaches for identification of candidate genes underlying these barriers, particularly among non-traditional model systems. We employ powerful proteomic and genomic strategies to study the genetic basis of conspecific pollen precedence, an important component of pre-zygotic reproductive isolation among yellow monkeyflowers (*Mimulus* spp.) resulting from male pollen competition. We use isotopic labeling in combination with shotgun proteomics to identify more than 2,000 male function (pollen tube) proteins within maternal reproductive structures (styles) of *M. guttatus* flowers where pollen competition occurs. We then sequence array-captured pollen tube exomes from a large outcrossing population of *M. guttatus*, and identify those genes with evidence of selective sweeps or balancing selection consistent with their role in pollen competition. We also test for evidence of positive selection on these genes more broadly across yellow monkeyflowers, because a signal of adaptive divergence is a common feature of genes causing reproductive isolation. Together the molecular evolution studies identify 159 pollen tube proteins that are candidate genes for conspecific pollen precedence. Our work demonstrates how powerful proteomic and genomic tools can be readily adapted to non-traditional model systems, allowing for genome-wide screens towards the goal of identifying the molecular basis of genetically complex traits.

## Author Summary

Barriers to reproduction are necessary for generating new species. Little is known about the genes underlying reproductive barriers, particularly those that function prior to fertilization, but their identity is of great interest as they offer insight into the genetic mechanisms and evolutionary forces generating biological diversity. In this work, we use an emerging plant model system for speciation studies (yellow monkeyflowers, species of *Mimulus*) to identify genes that might influence the relative competitive abilities of male pollen from the same versus different species within the maternal flower's style. This is a common reproductive barrier among plant taxa known as conspecific pollen precedence (CPP), and is analogous to sperm competition during animal fertilization. We first identify the pollen proteins that are found within the style where pollen competition occurs, and then screen these for evidence that may indicate which genes have been targets of pollen competition (a form of sexual selection among individuals of a population) or adaptive diversification among species of yellow monkeyflowers (a common feature of genes underlying reproductive barriers). Our evolutionary analyses identify 159 candidates that may function in reproductive isolation of yellow monkeyflowers, and provide some of the first broad perspectives on evolution of plant reproductive genes.

## Introduction

By identifying the genes underlying barriers to reproduction, we gain broad insight into the processes driving speciation and the origins of organismal diversity. Reproductive barriers have long been known to include multiple pre-zygotic mechanisms that function before fertilization, after which post-zygotic barriers including hybrid sterility and inviability mark an irreversible stage of reproductive isolation [1]. Significant progress has been made in understanding the mechanisms that underlie post-zygotic isolating barriers, and in several cases the causal genes have been identified [2]–[4]. This work has had tremendous impact on our understanding of the genetic basis and evolutionary forces underlying hybrid sterility and inviability, in particular highlighting the important role of genetic conflicts and epistatic genic incompatibilities known as Bateson-Dobzhansky-Muller (BDM) incompatibilities [2]. In contrast, significantly less is known regarding the genes underlying earlier acting pre-zygotic barriers. Of particular interest are barriers that result from divergence in traits and molecules that mediate mating or fertilization, and are thus likely targets of sexual selection. As with classical examples of sexually selected traits [5], sexual selection even at a molecular level can be a strong force leading to the rapid evolution of reproductive barriers [6].

Behavioral and morphological traits that function prior to mating, for example in courtship displays, have been of interest as targets of rapid divergence via sexual selection since Darwin [7]. Only more recently have the potential impacts of male competition, female preference, and sexual antagonism been widely recognized as extending to the host of morphological, physiological, and biochemical characters that impact reproductive success after mating but prior to fertilization [8]. Identifying the genetic basis of these post-mating, pre-zygotic reproductive barriers has proven challenging. Traditional genetic mapping approaches are complicated by the genetically complex nature of these isolating barriers, which typically reflect the small cumulative effects of many loci across the genome [9]. Biochemical characterization of their molecular basis is also technically difficult because they often involve competitive interactions among gametes which are manifest only within particular structures of the female reproductive system of animals or plants [10]. Instead, efforts have largely focused on indirect screens to distinguish reproductive genes without addressing their role in any specific post-mating barrier, for example by large scale sequencing of testis- or ovary-expressed genes [11], [12]. Then, because positive selection and rapid divergence are likely features of the genes underlying these reproductive barriers [13], comparative sequence data from closely related taxa or population polymorphism data can be analyzed using statistical approaches to distinguish candidates among reproductive genes [14]. More recently a number of biochemical and proteomic techniques have been developed to focus this work more discretely on particular reproductive structures or stages of reproduction during which reproductive barriers are known to arise (e.g., proteins of the egg coat [15] or accessory gland transferred to females during mating [16]). Such two-tiered approaches, incorporating characterization of constituent proteins in tandem with molecular evolutionary analyses to identify candidate genes, constitute an important step enabling targeted genetic mapping studies and ultimately functional characterization towards the ultimate goal of identifying the genetic basis of post-mating, pre-zygotic barriers.

The yellow monkeyflowers are an emerging model system for studies of the genetic basis of reproductive isolation and speciation in plants [17]. This group of taxa (*Mimulus guttatus* species complex [17], [18]) are ecologically and morphologically diverse but broadly interfertile [19], consistent with phylogenetic evidence of recent diversification [18]. Several large-flowered primarily outcrossing species are distributed across Western North America [20] including the widespread *M. guttatus* from which multiple self-pollinating taxa appear to be independently derived [21], [22]. Despite their recent divergence, populations and taxa are reproductively isolated by multiple pre-mating barriers (most notably flowering phenology [23], [24]) as well as conspecific pollen precedence (CPP [25]). This common post-mating but pre-zygotic barrier [25]–[34] results from conspecific male pollen out competing pollen from interfertile heterospecific taxa on the female pistil [10]. Among yellow monkeyflowers, CPP has been studied most extensively between *M. guttatus* and the closely-related selfer *M. nasutus*. These species have broadly overlapping ranges and hybrid individuals are frequent in some areas [19], [22], [24] with molecular evidence of introgression when they occur in sympatry [21]. Thus despite earlier acting premating barriers [23], [24], postmating barriers are important filters of genetic exchange between these taxa.

In interspecific crosses between *M. guttatus* and *M. nasutus*, CPP arises after unbiased germination on the stigma and is a unidirectional phenomenon whereby outcrossing *M. guttatus* pollen out competes pollen from the selfer within the outcrossing specie's styles, but is equivalent in siring success on the selfing maternal background [25]. Unidirectional CPP is consistent with theory [35], [36] showing sexual selection is manifest differently in taxa with different mating systems (differing degrees of outcrossing vs. selfing), demonstrating it's probable role in the evolution of this post-mating barrier between *M. guttatus* and *M. nasutus*. Significantly, analogous post-mating barriers among marine invertebrates (conspecific sperm precedence, CSP) present rare textbook examples of known speciation genes [37].

Conspecific pollen precedence between *M. guttatus* and *M. nasutus* is a genetically complex trait. Initial genetic mapping studies showed evidence of segregation distortion among many regions of an *M. guttatus×M. nasutus* F2 linkage map [38], for which the *M. guttatus* allele at mapped markers is predominantly in excess of Hardy-Weinerg expectations consistent with the observed pattern of unidirectional CPP. Reciprocal backcrosses of the F1 as both pollen and pistil parent to *M. guttatus* and *M. nasutus* [39] confirmed that: (*i*) segregation distortion favoring *M. guttatus* alleles at linked markers occurs only via the F1 pollen parent for at least 8 transmission ratio distortion loci (TRDL) distributed across the linkage map, consistent with the role of pollen competition causing the distortion; (*ii*) for the majority of these pollen-specific TRDL (7 of 8), distortion occurs only in the *M. guttatus* pistil background, consistent with observed unidirectional CPP in this cross; (*iii*) the effect size of any one pollen-specific TRDL is relatively small (*M. guttatus* allele favored 1.3 to 1.9-fold), but together they are sufficient to explain the near complete barrier CPP represents to *M. nasutus* pollen. Taken together, this is strong evidence that pollen-specific TRDL are regions that may contain candidate genes underlying CPP between *M. guttatus* and *M. nasutus*. These genes could also have a broader role in reproductive isolation and diversification of yellow monkeyflowers, as similar patterns of transmission distortion are known from crossing studies of *M. guttatus* with other members of the complex [40].

The goal of our work here is to identify candidate genes for CPP between *M. guttatus* and closely related taxa of yellow monkeyflowers, including *M. nasutus*. We focus specifically on identifying proteins of the male gametophyte (pollen tube) with evidence of positive selection and/or rapid adaptive divergence as providing an opportunity to link molecular screens and CPP via the expectation that pollen competition reflects one or more components of sexual selection — an expectation which specifically predicts sweeps and/or balancing selection for outcrossing populations of *Mimulus*, but which may also be reflected in a signal of adaptive divergence over longer time scales among taxa of yellow monkeyflowers. Earlier studies of pollen coat proteins in other species have suggested such positive selection could be a common feature of pollen proteins [41], though to our knowledge positive selection on genes with pollen tube function has been largely unexplored outside of self-incompatibility loci (though see [42]). Towards this goal, we first adapt a shotgun proteomic approach that utilizes isotopic labeling [16] in order to characterize genes with male function (the pollen tube proteome) within the style of pollinated flowers where pollen competition occurs. We then employ array capture to sequence complete pollen tube exomes from a large outcrossing population of *M. guttatus* to determine which genes are possible targets of directional or stabilizing selection. Finally, we test for evidence of long-term positive selection on these genes across yellow monkeyflowers. Our results provide a set of candidate genes that may underlie CPP for further investigation and represent one of the first broad perspectives of reproductive protein evolution from plants.

## Results

### Hydroponic cultivation and ^15^N labeling of *M. guttatus*

Cultivation of *M. guttatus* (IM62) plants in the hydroponic growth medium containing ^15^N KNO_3_ resulted in nearly complete labeling of style proteins. From high resolution mass spectra of pooled negative controls, the median level of ^15^N incorporation among peptides is 95% as estimated via Hardklör [43]. Moreover, protein identification from negative controls indicates essentially all style proteins are sufficiently ^15^N labeled to mask their detection using our shotgun proteomic techniques. Summing over reverse phase and MuDPIT analyses of pooled negative control styles from all three ^15^N labeled IM62 plants, only a single mass spectra was matched to each of 4 proteins, all of which were excluded from further analyses. Because we are able to match mass spectra for peptides corresponding to many thousands of proteins from pollinated styles using a similar level of MS/MS detection effort (see below), this is strong evidence supporting successful ^15^N labeling of maternal style tissues and allows us to confidently identify pollen tube proteins (PTPs) within the style.

### The *M. guttatus* pollen tube proteome

**Figure 1:**
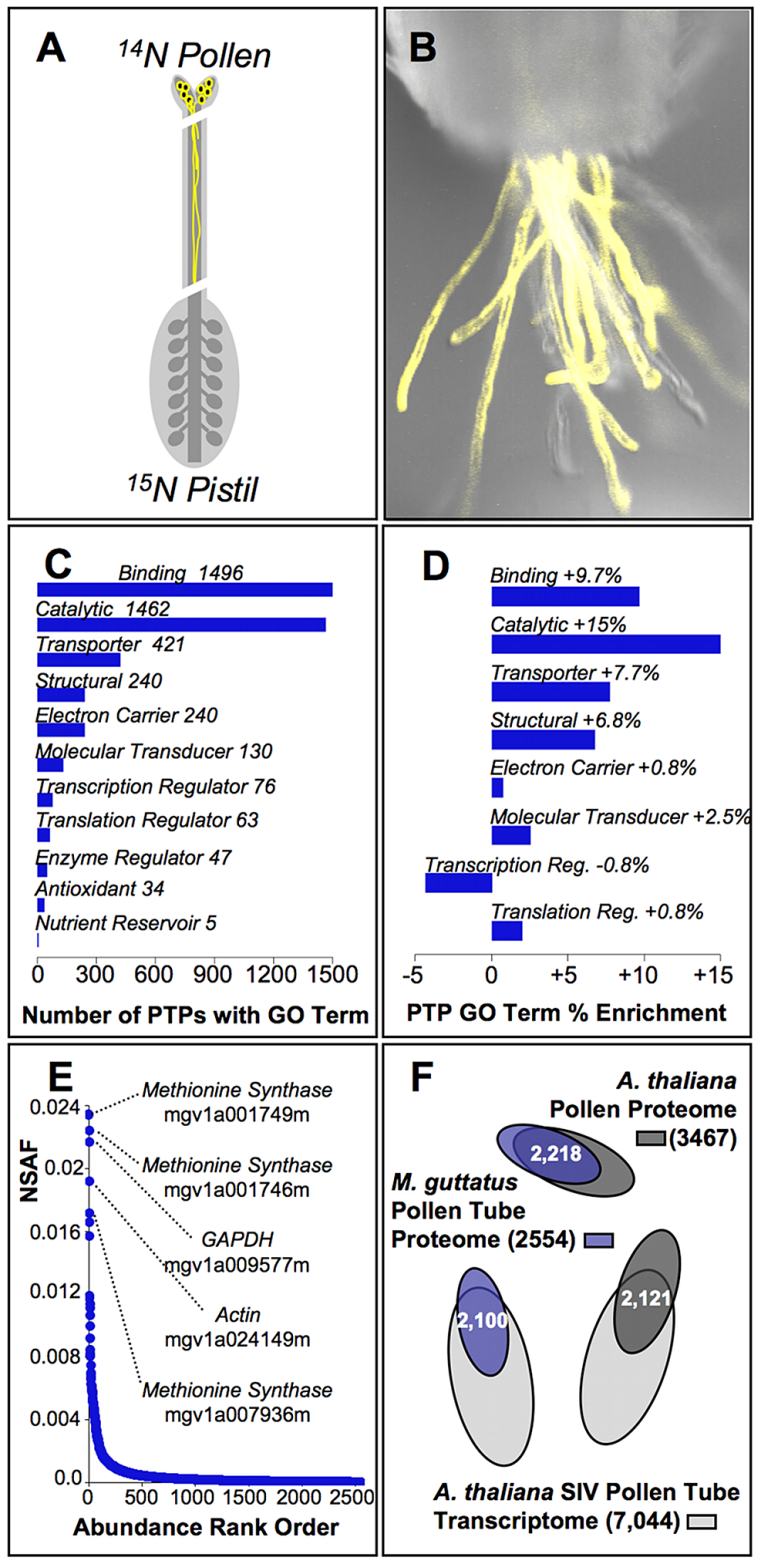
Characterization of the pollen tube proteome from yellow monkeyflowers. (A) The inbred strain of *Mimulus guttatus* (IM62) used for genome sequence construction was isotopically labeled with ^15^N via hydroponic culture and used as the pistil (maternal) parent in crosses to unlabeled (^14^N) IM62 pollen parents. Because ^15^N labeling masks maternal proteins in our MS/MS shotgun proteomic method, proteins can be identified as originating from *in vivo* pollen tubes unambiguously. (B) IM62 plants expressing a yellow fluorescent protein under the LAT52 pollen promoter (construct provided courtesy of Dr. Greg Copenhaver, UNC) emerge from a cut style. Pollen tube growth rate studies demonstrated pollen tubes grew approximately half the length of IM62 styles (∼8 mm) within 3 hours. (C) Of the 2,554 pollen tube proteins (PTPs) identified in our MS/MS studies, automated annotation [79] yielded 2,332 with associated molecular function GO terms (level 2 terms shown; see File S2 for a complete list). (D) GO terms from (C) that are significantly enriched among pollen tube proteins relative to the genome as a whole based on Fisher's exact test (p≤0.05). (E) Relative abundance of PTPs as estimated from the normalized spectral abundance factor (NSAF; File S1), covering a range of ∼3.5 orders of magnitude. The top five ranked accessions are identified along with their likely function. (F) Overlap among constituent genes of the *M. guttatus* pollen tube proteome (blue, this study), *Arabidopsis thaliana* whole pollen proteome [red, [45]], and *A. thaliana* pollen tube transcriptome [green, [46]].

The pollen tube proteome of *M. guttatus* consists of more than 2,000 proteins. Mass spectra from ^15^N labeled styles pollinated with unlabeled (^14^N) pollen (Fig. 1A) were matched to 2,554 protein coding transcripts from the *M. guttatus* genome (File S1). More than 81% of these (2,073) are distinguished by unique peptides, resulting in unambiguous protein identification. The remainder (481) are identified from peptides common to two or more proteins, and are thus designated as members of (arbitrarily numbered) multi-protein groups that may include one or more member proteins (File S1). Overlap in protein identification among replicate (independently ^15^N labeled) IM62 plants is substantial but incomplete, with ∼60% of PTPs shared between any two and identification of 36% common among all three replicates (Fig. S1). The majority of PTPs (97%) were identified from MuDPIT analyses with about half of PTPs identified in our reverse phase analyses despite similar total MS/MS scan time for both methods (72 versus 60 hours, respectively). Significantly, because ^15^N labeling effectively masks maternal stylar peptides and we used conservative filters for matching of peptides from ^14^N pollen (≥1 unique peptide per protein, q-value≤0.002), PTP identifications are made with high confidence. The MS/MS spectra and sequest search results for all analyses are publicly available (https://sites.google.com/a/uw.edu/maccoss/home/supplementary_data/).

Functional annotation of PTPs shows the pollen tube proteome of *M. guttatus* is enriched for several classes of proteins. Among the 2447 PTPs successfully annotated (96% of the proteome), gene ontology (GO) terms associated with rapid growth and metabolism are the most frequent functional classifications (Fig. 1C; File S2), and are significantly enriched among PTPs relative to the genome as a whole (Fig. 1D). Proteins sharing these classifications are also among the most abundant PTPs within pollinated styles, their relative abundance (NSAF) more than 3 orders of magnitude higher than the median value (Fig. 1E; File S1). Interestingly, despite extensive divergence since the last common ancestor between *Mimulus* and *Arabidopsis* (∼120 million years ago) [44], gross protein sequence similarity (e-value≥e^−20^) with *A. thaliana* pollen proteins [45] and pollen tube transcripts [46] includes 85% on average of the proteins we identified from *M. guttatus* pollen tubes (Fig. 1F).

### Many PTPs are not unique to the pollen tube

Mass spectra from unpollinated, unlabeled (^14^N) styles were matched to 2,608 protein coding transcripts from the *M. guttatus* genome (File S1). More than 3/4 of these proteins (1,988) are distinguished by unique peptides, resulting in unambiguous protein identification, while the remainder (620) are identified from peptides common to two or more proteins (multi-protein groups). Of the 2,554 pollen tube proteins we identified, 51% are also found in unpollinated styles of *M. guttatus* (1,310; Fig. S1C, File S1). Thus ∼1/2 of all PTPs are not unique to the male gametophyte, and can found at high relative abundance (File S1) in female structures of the flower.

### Selection among PTPs at Cone Peak

**Figure 2:**
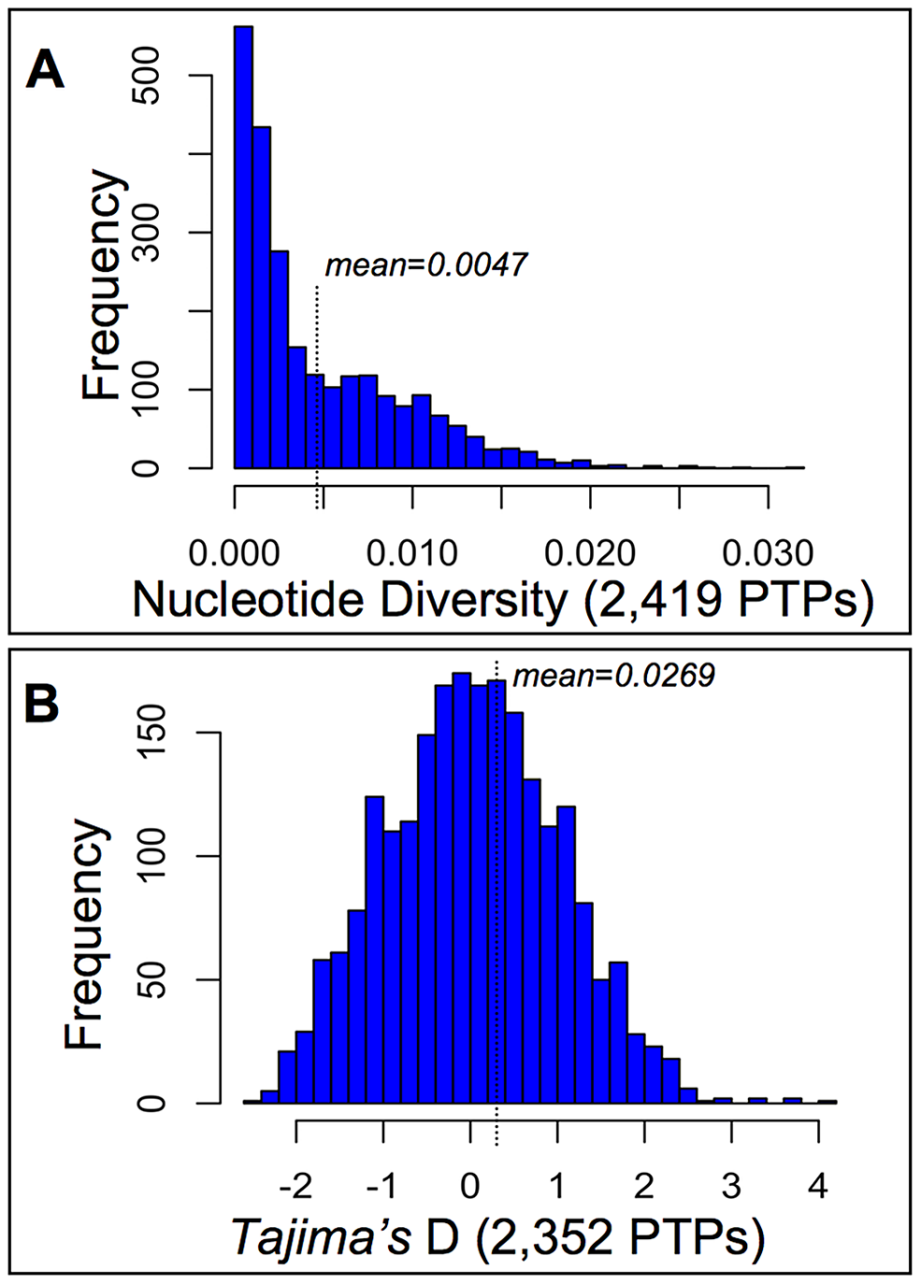
Diversity and selection among pollen tube proteins (PTPs) from a natural population of *Mimulus guttatus*. PTP Exomes of 28 wild collected individuals from the Cone Peak population of *M. guttatus* [81] were captured on a custom Agilent micro-array and Illumina sequenced, after which reads were assembled and mapped to IM62 CDSs. Base calls were made for sites with ≥10 reads, from which measures of nucleotide diversity per site (expected heterozygosity) and *Tajima's* D [64] were made after excluding sites with missing data and those that violate the infinite sites model [88]. (A) Our SNP calling procedure identified ∼1.3×10^6^ variants (of 3.4×10^6^ total bases called after filtering), with at least one SNP called for 97% of successfully captured CDSs (2,419; File S3) yielding 0.47% of a PTPs coding sequence on average polymorphic. (B) The distribution of *Tajima's* D values calculated from PTPs harboring SNPs (2,352; File S3) shows no evidence of confounding demographic forces at Cone Peak (mean D = 0.0269), thus the upper and lower tails of the distribution (2.5%) were chosen as constituting a 95% confidence interval beyond which PTPs may be targets of balancing selection or sweeps, respectively.

Our array enrichment and sequencing of exomes from 28 *M. guttatus* plants collected at Cone Peak yielded population polymorphism data for ∼95% of the PTPs we identified in our MS/MS studies (2,419; File S3). Of 3.4×10^6^ bases called after filtering raw reads, ∼1.3×10^6^ variants were identified, with at least one SNP called for 97% of PTPs captured that identify 0.47% of a PTP's coding sequence polymorphic on average (Fig. 2A). The raw Illumina reads from array-captured pollen tube exomes of Cone Peak *M. guttatus* are publicly available (NCBI sequence read archive accession SRP029938).

The distribution of *Tajima's* D values calculated from PTPs harboring polymorphism (2,352) shows no strong evidence of confounding demographic forces at Cone peak (Fig. 2B), though the distribution is somewhat long-tailed towards positive values, resulting in a mean slightly greater than zero (mean D = 0.0269). The 2.5% tails of the distribution correspond to *Tajima's* D values less than −1.83 and greater than 1.99 (File S3), beyond which we interpret PTP's *Tajima's* D statistic as showing evidence of sweeps or balancing selection, respectively. As confirmation that these thresholds of the empirical distribution constitute a 95% confidence interval under the neutral expectation, we simulated data under a neutral coalescent model for each of the 116 PTPs in the tails — in all cases p-values from simulations were ≤2.5% (File S3), consistent with the empirical distribution and in support of either sweeps or balancing selection acting on these genes. For the 58 PTPs with evidence of sweeps based on *Tajima's* D, we were able to identify orthologs from *M. tilingii* or *M. cupriphilus* for 56, from which *Fay and Wu's* H was calculated. Calculations using *M. tilingii* as an outgroup identify 36 of these as targets of sweeps based on statistical support from coalescent simulations (p≤0.05), similar to results using *M. cupriphilus* as an outgroup (39 PTPs; File S3). Based on a significant test statistic using one or more of these taxa as outgroups, *Fay and Wu's* H validates the inference of sweeps for 44 of the 58 PTPs identified via *Tajima's* D (76%), consistent with the view that population demographic history at Cone Peak does not confound inferences of selection via statistical tests based on site frequency spectra.

### PTPs evolve under strong constraint among *Mimulus* taxa

**Figure 3:**
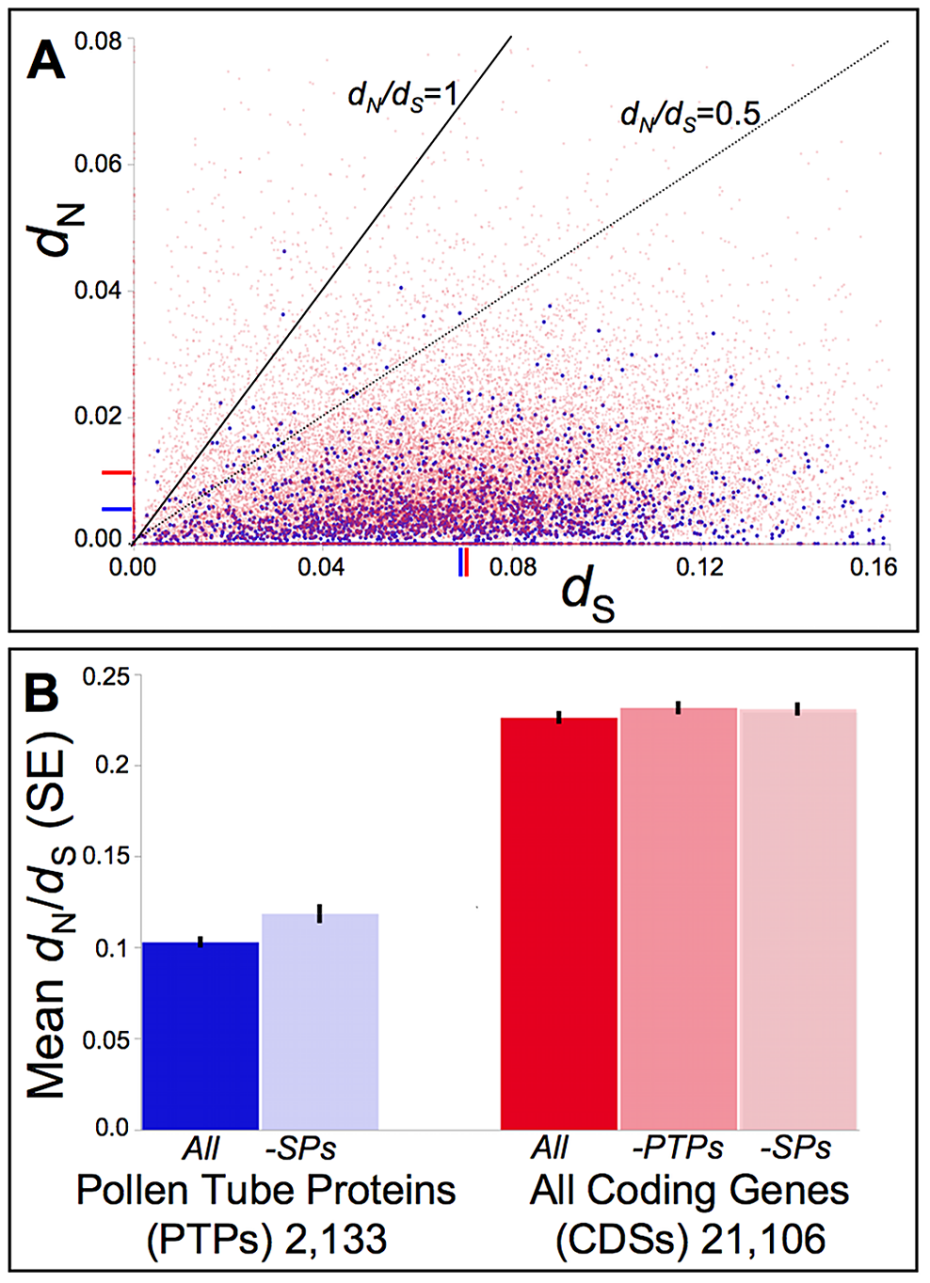
Distribution of pairwise non-synonymous (*d*_N_) and synonymous (*d*_S_) nucleotide substitutions as well as their ratio (*d*_N_/*d*_S_, or ω) among *Mimulus* pollen tube proteins (PTPs) and coding sequences (CDSs) genome-wide. (A) Putative orthologs of *M. guttatus* (IM62) genes from *M. tilingii* [an outgroup to the *M. guttatus* species complex [17], [18]] were identified via mapping and assembly of the Illumina sequenced STV115 library (NCBI sequence read archive SRX142376). Pairwise measures of *d*_N_ and *d*_S_ were calculated for putative orthologs via codeml in the PAML computer package [93]. The distribution of pairwise *d*_N_ -vs- *d*_S_ values relative to benchmarks of *d*_N_/*d*_S_ = 1.0 (neutral expectation) and *d*_N_/*d*_S_ = 0.5 demonstrate strong selective constraints act on the majority of PTPs (blue) as well as the remaining CDSs (red), though a small subset show evidence consistent with positive selection (*d*_N_/*d*_S_>1.0). Mean *d*_N_ and *d*_S_ values are indicated on the respective axes for PTPs (blue) or CDSs genome-wide (red). While the mean *d*_S_ does not differ significantly between PTPs and other CDSs (0.067 vs. 0.07, respectively; p = 0.69), the mean *d*_N_ for PTPs (0.005) is 2-fold lower than for other CDSs (0.011; p<0.001). (B) Putative orthologs of IM62 CDSs from a dune ecotype of *M. guttatus* and 8 additional taxa of the *M. guttatus* species complex (*M. cupriphilus*, *M. dentilobus*, *M. glaucescens*, *M. micranthus*, *M. nasutus*, *M. nudatus*, *M. pardalis*, and *M. platycalyx*) were identified via mapping and assembly of other available Illumina sequenced libraries (SRX030540-1, SRX030973-4, SRX116529, and SRX142372-5). For each CDS alignment, ω was estimated via a single-ratio model (M0) in codeml using CDS-specific topologies for categories of PTPs, style proteins (SPs), and CDSs generally (see Materials and Methods). On average, PTPs evolve under stronger constraint than other CDSs genome-wide [mean *d_N_*/*d_S_* = 0.10 vs. 0.23 for PTPs and all other CDSs (-PTPs), respectively; p<0.01], even after removing those PTPs also found in our MS/MS studies of style proteins which may constitute a class of general housekeeping or developmental regulatory genes (*d_N_*/*d_S_* = 0.11 after removing SPs; p<0.01).

Our assembly and mapping of publicly available Illumina reads for the outgroup *M. tilingii* yielded pairwise estimates of synonymous (*d*_S_) and non-synonymous (*d*_N_) substitution rates from alignment with IM62 protein coding sequences (CDSs) for 25,550 CDSs genome wide, including 2,262 PTPs (Fig. 3A). While the range of *d*_S_ values among PTPs is comparable to other CDSs (0.067 vs. 0.070, respectively; p = 0.69), non-synonymous substitution rates (*d*_N_) are markedly lower (0.005 vs. 0.011, respectively; p<0.001). This pattern of strong evolutionary constraint among PTPs is further supported by our mapping and assembly of publicly available Illumina reads for the 9 additional accessions of the *M. guttatus* species complex, which yielded *d*_N_/*d*_S_ estimates from the one-ratio model M0 for 21,106 CDSs genome wide, including 2,133 PTPs (Fig. 3B). Mean *d_N_*/*d_S_* for PTPs was less than half the genome-wide average for other CDSs (0.10 vs. 0.23 for PTPs and all other CDSs (-PTPs), respectively; p<0.001] even after removing those also found in our MS/MS studies of style proteins [mean *d_N_*/*d_S_* = 0.11 for PTPs after removing SPs; p<0.001 for comparison with other CDSs (-SPs)].

**Figure 4:**
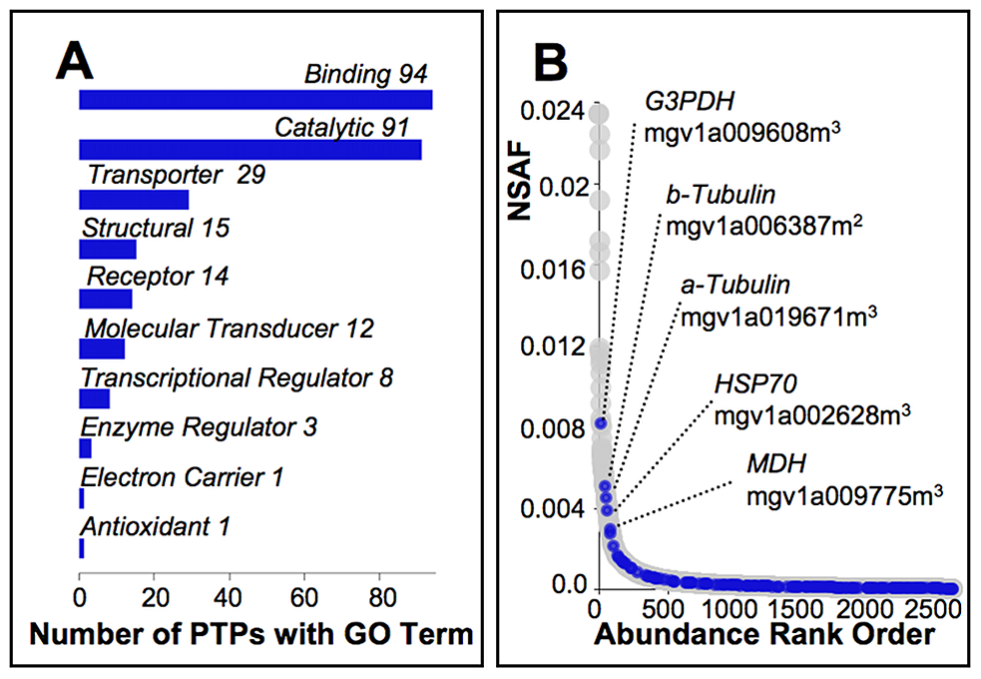
Characterization of pollen tube proteins targeted by selection. (A) Of the 159 PTPs identified in our molecular evolution studies as showing a signature of selection consistent with adaptive divergence among *Mimulus* species, or selective sweeps or balancing selection among Cone Peak *M. guttatus*, automated annotation [79] yielded 151 with associated molecular function GO terms (level 2 terms shown), none of which are significantly under or over represented relative to other PTPs. (B) Relative abundance of the 159 PTPs targeted by selection (blue ovals) as inferred from the normalized spectral abundance factor (NSAF) span the range for PTPs as a whole (grey ovals; File S1). The five most abundant include those with likely roles in pollen tube metabolism, stress response, or that have structural functions, which exhibit evidence of selective sweeps (^2^) or balancing selection (^3^) at Cone Peak.

While strong evolutionary constraints characterize divergence of PTPs as a whole, sites models of codon evolution identify a small subset (∼2%) with evidence of positive selection among yellow monkeyflowers (File S3). Manual validation of computational alignments for the 45 PTPs for which likelihood ratio tests pass the multiple-comparison corrected significance threshold (q≤0.10) confirmed statistical support for all but two (File S3). Only one of these genes also shows evidence of adaptive diversification in the *M. guttatus* population at Cone Peak (mgv1a018842m; File S3), which is not a significant enrichment relative to PTPs as a whole (one-tailed p-value = 0.327 from Fisher's exact test). Functional classifications of the 43 PTPs with evidence of adaptive divergence in combination with the 116 with evidence of sweeps or balancing selection at the Cone Peak population of *M. guttatus* are similar to PTPs as a whole (Fig. 4A). Fisher's exact test identifies no GO categories as significantly enriched relative to other PTPs. In addition, their relative abundances (NSAF) span the range of values calculated for PTPs as a whole (Fig. 4B), consistent with selection acting on PTPs independent of protein abundance, and nearly half these genes (48%) were also identified in our MS/MS studies of style proteins, suggesting they are not enriched for pollen-specific functions.

## Discussion

We employ proteomic characterization of the *M. guttatus* pollen tube in combination with molecular evolution studies towards the goal of identifying candidate genes for conspecific pollen precedence (CPP), a post-mating but pre-zygotic isolating barrier between *M. guttatus* and *M. nasutus*. We first characterized more than 2,000 proteins using a ^15^N labeling approach that allows for *in vivo* identification of pollen tube proteins (PTPs) within the style where pollen competition underlying CPP manifests. Then, because sexual selection acting at the population level is the likely evolutionary force responsible for this phenomenon, we tested for evidence of selective sweeps or balancing selection among PTPs in a large outcrossing population of *M. guttatus* using array capture of pollen tube exomes. We also tested for positive selection on PTPs more broadly among taxa of yellow monkeyflowers because a signal of adaptive divergence is often characteristic of genes underlying reproductive barriers. We summarize several important results from the proteomic and molecular evolution screens of pollen tube genes that individually add to our basic knowledge of the genetic basis of plant reproduction [47]–[50] and to our understanding of the evolution of reproductive molecules [13], [51]. Finally, we present a brief summary of candidate genes for future targeted studies of CPP in *Mimulus*.

### Pollen tube proteomics

The *M. guttatus* pollen tube proteome is a significant contribution to plant reproductive biology that: (*i*) demonstrates the broad applicability of *^15^N* labeling for distinguishing male and female reproductive proteins *in situ*; (*ii*) catalogs male reproductive genes relative to model plant species such as *Arabidopsis*; and (*iii*) represents a significant increase over previous proteomic work, but is nonetheless likely to be a conservative picture of the proteins present in growing *M. guttatus* pollen tubes.

### ^15^N labeling provides a new tool for plant reproductive biology

To identify pollen tube proteins (PTPs), we adapted a novel isotopic labeling scheme in order to characterize the pollen tube proteome within the particular female structures of the flower (styles) where pollen competition occurs. We grew plants used as the female pistil parent on hydroponic media containing ^15^N KNO_3_ as the sole source of nitrogen [52], which rendered them effectively “invisible” in our MS/MS-based proteomic analyses. By using unlabeled (^14^N) plants as pollen donors in controlled pollinations, we are able to identify and characterize proteins within ^15^N styles that were unambiguously derived from pollen tubes (Fig. 1A). The general approach was recently developed in *Drosophila* to identify male seminal fluid proteins within the female reproductive tract of flies [16], but had not been applied to plants previously. Our success with ^15^N labeling in a plant system demonstrates the approaches potential broad utility for distinguishing among male and female reproductive proteins *in situ*.

### The *M. guttatus* pollen tube proteome complements investigations in model taxa

Our catalog of the molecular components of pollen tube growth and guidance within the pistil (File S1) complements expression data [46], [53], [54] and smaller proteomic studies [45], [55]–[57] from other plant taxa. Shotgun proteomic analyses unambiguously identified 2,073 PTPs from unique peptides found within the style of pollinated ^15^N-labeled pistils of *M. guttatus*. An additional 481 correspond to multi-protein groups consisting of one or more member proteins with shared peptides. The majority of these 2,554 proteins (File S1) are characterized by GO annotation terms (Fig. 1C) consistent with their role as components of the molecular machinery for rapid semi-autonomous growth and guidance of pollen tubes through the maternal style [47]–[50] and that are enriched in the pollen tube proteome (Fig. 1D, File S2). For example, among the most abundant are several methionine synthases (Fig. 1E) that have previously been identified as pollen tube components in other plant taxa [55], [58], likely facilitating targeted pollen tube tip growth through their role in diverse metabolic pathways [59], [60]. Many of these genes are likely to also be expressed in other tissues (see below), including structures of the female pistil: our MS/MS studies demonstrate that ∼1/2 of PTPs can also be found within unpollinated styles (Fig. S1C, File S1). While the large number of PTPs identified in *Mimulus* precludes detailed discussion, many of these genes appear to have homologs in model plant taxa as evidenced by the large degree of overlap with the *A. thaliana* whole pollen proteome [45] and pollen tube transcriptome [46] (Fig. 1F). This overlap emphasizes the many rich opportunities for more detailed comparative studies of plant reproduction between established model systems and emerging model systems like *Mimulus* that represent distinct evolutionary lineages, and for which genomic and proteomic tools are rapidly developing.

### *M. guttatus* PTPs are a conservative representation of the pollen tube proteome

The number of PTPs we identified *in situ* from *M. guttatus* (>2,000) is remarkable when compared with similar studies of animal reproductive proteins. For example, we identified ∼10-fold more proteins than previous work employing the same ^15^N-labeling approach with similar MS/MS-based protein detection methodologies in *Drosophila* [16]. This increase undoubtedly reflects the greater biological complexity of the male gametophyte of flowering plants, though variation in MS/MS detection effort, degree of maternal ^15^N labeling, or genome annotation may also contribute. Like a recent study of *Arabidopsis* whole pollen employing comparable methodologies [45], our work represents a >3-fold increase over previous (principally 2-D gel based) proteomic studies of pollen and pollen tubes [55]–[57]. However, the PTPs we have identified in *M. guttatus* are still likely to be a conservative representation of the pollen tube proteome for several reasons. First, we reduced power to detect proteins [61] by using conservative protein identification filters aimed at minimizing the false discovery rate. Second, peptides from PTPs represent a small fraction (∼8%) of the analyzed mass spectra from pollinated styles. Thus, many additional PTPs could be masked by the disproportionate abundance of ^15^N-labeled peptides from stylar proteins, as suggested by the relatively low overlap in proteins identified among biological replicates (∼60% between any two; Fig. S1). PTPs could also be masked if transcripts were translated *in vivo* from amino acid residues derived from the maternal (^15^N) stylar environment. Third, *Arabidopsis* pollen expresses >7,000 transcripts, a large proportion of which are unique to the pollen tube [46]. Although the typically low correlation between transcript and protein abundance argues against a one-to-one correspondence of the underlying loci [62], this again suggests that additional *Mimulus* PTPs await characterization.

### Evolutionary screens of *Mimulus* Pollen Tube Proteins (PTPs)

Our studies of the molecular evolution of *M. guttatus* PTPs: (*i*) identify the loci that may be targets of selective sweeps or balancing selection in a large outcrossing population of *M. guttatus* where sexual selection can be a potent evolutionary force on reproductive genes; (*ii*) test for the action of positive selection more broadly in adaptive divergence of PTPs among closely related members of the *M. guttatus* species complex; and (*iii*) address questions regarding rates of evolution among male plant reproductive genes in a broader genome-wide context.

### Targets of selection among *M. guttatus* at Cone Peak

Our analyses of array-captured *M. guttatus* PTP exomes from Cone Peak identify the subset of these genes that are likely targets of selective sweeps or balancing selection. Coding regions of the 2,419 PTPs we were able to sequence via array capture harbor relatively high levels of polymorphism (Fig. 2A), comparable to silent site diversity for a much smaller set of loci within natural populations of *Arabidopsis lyrata* [63], one of the few outcrossing taxa with data available for comparison with our results. However, unlike previously studied populations of *A. lyrata*, the distribution of T*ajima's D* statistics calculated from *M. guttatus* PTPs harboring polymorphism at Cone Peak (Fig. 2B) is very close to that expected under theory for an ideal population in the absence of demographic forces such as bottlenecks [64]. Because we've assayed SNPS at ∼10% of IM62 CDSs genome-wide, this suggests demographic factors have not been a strong evolutionary force at Cone Peak and that the empirical distribution of *Tajima's D* can serve to identify PTPs that deviate from the expectation under neutrality. The significance threshold (α = 0.05) set from the 2.5% tails of the empirical distribution and validated by coalescent simulations (see File S3) identify 116 genes in total for which neutrality is rejected, corresponding to 58 PTPs with evidence of selective sweeps and 58 with evidence of balancing selection (negative and positive *D*, respectively; File S3). For the subset of PTPs inferred to be targets of selective sweeps, *Fay and Wu's* H provides further support for positive selection acting on 44 of these PTPs at Cone Peak. Significantly, the high degree of overlap (76%) between these two complementary tests of selection utilizing different portions of the site frequency spectra that are more (*Tajima's* D) or less (*Fay and Wu's* H) subject to the influence of population growth also supports the view that our results are largely free from the influence of demographic history.

Sweeps or balancing selection on these 116 PTPs are consistent with the action of sexual selection manifest among outcrossing *M. guttatus* plants at Cone Peak. Sexual selection could result most simply from a strictly male-male competition scenario, in which the haploid genotype of the pollen tube is the sole determinant of male performance. A straightforward prediction of this scenario is selective sweeps at the underlying loci in outcrossing *M. guttatus* populations such as Cone Peak. Alternately, pollen tube growth via maternal guidance mechanisms and molecules produced within the style and translocated to the pollen tube [47]–[50] provides the opportunity for direct female mediation of male-male competition (female choice) and for antagonistic coevolution between male and female (sexual conflict) [36]. Both forms of sexual selection are likely to be relaxed in selfers such as *M. nasutus* [35], but should exert strong pressure in outcrossing *M. guttatus* populations such as Cone Peak. Under some female-choice scenarios, determinants of pollen function may be under balancing selection in outcrossers, whereas other scenarios predict frequent selective sweeps [35], [36]. And because sexual conflicts may resolve differently, the underlying genes can be under either directional or balancing selection among different populations, a complex pattern known for fertilization proteins important in conspecific sperm precedence (CSP) [65].

Genes under sexual selection within outcrossing populations such as Cone Peak could give rise to reproductive barriers among divergent members of the *M. guttatus* species complex including *M. nasutus* via several mechanisms. First, if alleles are under sexual selection due simply to male-male pollen competition, faster growing pollen tubes carrying these alleles should outcompete heterospecific pollen from taxa lacking a history of strong pollen competition and resulting in a higher frequency of conspecific fertilizations. In the absence of more complex genetic interactions (see below), this constitutes a strong barrier only between outcrossers such as *M. guttatus* and primarily self-pollinating taxa such as *M. nasutus* [25] and predicts that alleles from outcrossers can rapidly invade populations of self-pollinating taxa. There is good evidence that at least some of the transmission ratio distortion loci (TRDL) which contribute to unidirectional CPP between *M. guttatus* and *M. nasutus* harbor such simple pollen performance genes based on reciprocal backcrosses demonstrating distortion acts independent of the maternal style's genotype for these loci [39]. Because other members of the complex are also likely to experience lower rates of outcrossing than *M. guttatus* [66], they could be a potent unidirectional barrier limiting gene flow.

Second, if allelic variation for female pistil genes does influence the outcome of pollen competition, reproductive incompatibilities can arise between populations or taxa harboring divergent alleles at interacting reproductive genes as a form of Bateson-Dobzhansky-Muller (BDM) incompatibility. Sperm and egg fertilization proteins from marine invertebrates are well documented examples of this form of reproductive barrier [13] which constitute rare textbook examples of speciation genes [37]. Importantly, because cognate fertilization genes must co-evolve to maintain compatibility among interbreeding individuals within a population, their divergence driven either as a result of female preference or sexual conflicts can result in reproductive barriers among divergent populations or taxa which function without respect to mating system. Consistent with the idea that they may function in reproductive isolation between *M. guttatus* and *M. nasutus*, reciprocal pollinations show that *M. nasutus* styles do not support rapid growth of *M. guttatus* pollen tubes [25] and distortion for a subset of the TRDL underlying CPP between *M. nasutus* and *M. guttatus* is style specific [39] suggesting they harbor female modifiers of male function alleles expressed by pollen tubes.

### Adaptive divergence of PTPs across the *M. guttatus* species complex

We also tested for positive selection on PTPs more broadly among representative yellow monkeyflowers for which raw genomic sequence reads were publicly available. These include a dune ecotype of *M. guttatus* along with 8 additional taxa of the *M. guttatus* species complex (*M. cupriphilus*, *M. dentilobus*, *M. glaucescens*, *M. micranthus*, *M. nasutus*, *M. nudatus*, *M. pardalis*, and *M. platycalyx*) and a closely related outgroup, *M. tilingii*. Of the 2,133 PTPs meeting our criteria for assembly, mapping, and codeml analyses (see Materials and Methods), ∼2% show evidence of adaptive divergence under positive selection. There is almost no overlap between these 43 genes and the 116 with evidence of sweeps or balancing selection at Cone Peak – only one (mgv1a018842m; File S3) is identified as under balancing selection as well as evolving under positive selection among yellow monkeyflowers, which is not a significant enrichment relative to PTPs as a whole (one-tailed p-value = 0.327 from Fisher's exact test). The different sets of genes identified by these analyses are most likely due to the very different time scales for which the tests we employ have power to detect signatures of selection [67]. However, we cannot exclude the possibility that evolutionary forces acting among yellow monkeyflowers are largely distinct from those that function within populations, e.g., reflecting selection pressures of pathogens as opposed to recurrent sexual selection among populations. Regardless, because a signature of positive selection from phylogeny-based tests of *d*_N_/*d*_S_ ratios is a well established feature of the genes underlying barriers to reproduction [13], we consider PTPs with evidence of adaptive divergence as additional candidates that may contribute to reproductive isolation between *M. guttatus* and *M. nasutus*, and perhaps more broadly among yellow monkeyflowers.

### *Mimulus* PTPs are a slowly evolving class of protein coding genes

We find that constituent proteins of the male gametophyte of yellow monkeyflowers evolve under markedly stronger evolutionary constraint than most other protein coding sequences in the genome (Fig. 3A,B). Non-synonymous substitutions (*d*_N_) for 2,262 PTP orthologs average only about half that calculated for the remaining 23,288 protein coding sequences (CDSs) calculated from pairwise differences between *M. guttatus* and *M. tilingii* (0.005 -vs- 0.011, respectively; p<0.001) despite comparable levels of synonymous substitution (*d*_S_ = 0.067 and 0.070, respectively; p = 0.069). Similarly, *d*_N_/*d*_S_ ratios averaged across sites (model M0) of 2,133 PTPs for which we were able to map and assemble orthologs from multiple members of the *M. guttatus* species complex are less than half that of the remaining 18,973 CDSs (*d*_N_/*d*_S_ = 0.10 and 0.23, respectively; p<0.001). This pattern persists when we remove from the comparison those CDSs that are shared between both pollen tube and style proteomes (Fig. 3B).

Our finding that genes with male function in plants evolve under strong constraint is in contrast with longstanding evidence across animal groups that male function genes evolve more rapidly than others [68]. For example, genes expressed specifically in male reproductive tissues or with male-biased expression are among the fastest evolving protein coding sequences between the *Drosophila melanogaster* and *D. simulans* lineages [69]. Previous smaller scale studies of pollen genes finding evidence of rapid divergence under positive selection (e.g., [70]) had hinted that similar trends might be found for genes with male function in plants. While we too find rapid adaptive divergence for a small subset of PTPs, at least in *Mimulus* genes with male function clearly do not fit the pattern predicted from animal studies.

In part, this may simply reflect the greater biological complexity of pollen tubes. The male gametophyte represents a distinct life-history stage of plants that is semi-autonomous with respect to many aspects of cellular growth and respiration [49], [59], [60], suggesting a higher proportion of PTPs could be constrained due to essential house-keeping or developmental regulatory function. Importantly, the evolutionary dynamics of haploidy versus diploidy [71] could contribute strongly to this effect. Unmasking of recessive alleles during pollen tube growth in competitive pollinations should result in more rapid purging of slightly deleterious non-synonymous substitutions for genes expressed in haploid male gametophytes, consistent with lower levels of *d*_N_ and smaller *d*_N_/*d*_S_ ratios for PTPs relative to genome-wide averages (Fig. 3A,B). Under this scenario, slower rates of evolution for plant male function genes reflect the efficiency of selection acting during the male gametophytic stage as opposed to greater essentiality or lower potential for positive selection *per se* relative to male function genes in animals.

### Conspecific Pollen Precedence (CPP) candidate genes

We identify a total of 159 PTPs that are candidate CPP genes based on theory and empirical evidence identifying sexual selection within outcrossing *M. guttatus* as the likely driving force of pollen competition in crosses with *M. nasutus*, and divergence under positive selection as a common feature of genes underlying reproduction among closely related species such as members of the *M. guttatus* species complex (File S3). As with PTPs generally (Fig. 1C,D), many of these candidates appear to function in basic aspects of cellular growth and metabolism, with the majority yielding GO terms associated with binding or catalytic activity (Fig. 4A). Interestingly, because there is no evidence of GO term enrichment among candidates and they span the range of relative protein abundances (NSAF) observed among PTPs generally, their distinguishing features appear to be limited to evidence of sweeps, balancing selection, and adaptive divergence.

To our knowledge, pollen tube phenotypes have been identified in other plant model systems for homologs of just three of these 159 PTPs. These include an *Arabidopsis thaliana* homolog (AT1G08660) of mgv1a005647m, a glycosyl transferase for which mutants are known to show reduced pollen tube germination and growth rates [72], along with an *A. thaliana* homolog (AT1G14420) of mgv1a006379, a pectate lyase-like gene first characterized in tomato (LAT59) and notable for studies of it's pollen-specific promoter element [73]. In addition, they include an *M. guttatus* homolog (mgv1a000017m) of *A. thaliana* KINGY POLLEN (KIP; AT5G649680) [74]. KIP is a large secreted protein, and mutants exhibit periodic growth arrest followed by axis reorientation, thought to be due to disruption of secretory trafficking at the Golgi membrane based on studies of homologs in corn [75], where it is known to influence pollen tube growth rates and pollen competition.

### Future work on candidate PTPs

Because the pollen-specific TRDLs underlying CPP between *M. guttatus* and *M. nasutus* manifest as quantitative effects on genome transmission in F1 hybrids and backcross populations, and often exhibit broad peaks [39], their characterization by positional cloning would be extraordinarily laborious. Instead, we can now ask a relatively straightforward question in the context of higher resolution segregation studies focusing on our 159 candidate genes: *does a candidate gene exhibit more extreme transmission distortion in F2 and backcross populations of M. guttatus×M. nasutus than increasingly distant flanking genes, and is this distortion pollen-specific?* If a given candidate is at the core of a region of local pollen-specific distortion, reverse genetic confirmation of its effects on transmission can rapidly follow as part of future studies. Thus our screen for candidate genes promises to accelerate the molecular characterization of the many loci underlying a complex, common, and poorly understood species barrier in flowering plants.

### Conclusions

The increasing availability of genomic data from non-model taxa such as yellow monkeyflowers presents opportunities to unravel the complex genetic basis of pre-zygotic reproductive barriers. However, bringing this data to bear on questions of reproductive isolation can be daunting given the complexities of linking variation in genomic-scale data with particular reproductive barriers that have a complex genetic basis. We demonstrate one approach to this challenge by first identifying the constituent proteins of the *M. guttatus* pollen tube within the maternal style using ^15^N labeling and shotgun proteomics – these male function genes include the subset of loci responsible for pollen competition underlying conspecific pollen precedence (CPP) between *M. guttatus* and the sister taxon *M. nasutus*. Then, because sexual selection within outcrossing *M. guttatus* populations and adaptive divergence among closely related members of yellow monkeyflowers are expected features of the genes contributing to CPP, we test for these signals proteome-wide. Using this approach allows us to identify 159 candidate genes for CPP, a common reproductive barrier between closely related plant taxa including species of *Mimulus*. Though several of these genes have known functions or exhibit mutant phenotypes in model species that may be of relevance to pollen competition, the challenge now is to explicitly test their role in reproductive isolation via fine-mapping of pollen-specific distortion around candidates followed by functional and genetic confirmation.

## Materials and Methods

### Isotopic labeling of *M. guttatus* to identify pollen tube proteins *in vivo*

An inbred strain of *M. guttatus* (IM62) used previously for construction of genetic linkage maps [38], [40] and genome sequencing [17] was isotopically labeled using a modified hydroponic cultivation method. IM62 seeds were germinated on potting soil, and after two weeks transferred to polystyrene plugs placed in 0.5 L aerated containers filled with hydroponic media which contains ^15^N-potassium nitrate (KNO_3_; Cambridge Isotope Laboratories, Andover, MA) as the sole source of nitrogen, as in [52]. Plants were grown on ^15^N media until they flowered (approx. 12–16 weeks post germination) and used as pistil parents in crosses to unlabeled (^14^N) IM62 plants. For crosses, unlabeled pollen was collected from 40 flowers 2–3 days post anthesis and used to hand pollinate 30 virgin flowers on each of 3 separately ^15^N labeled IM62 plants (full replicates). Pollinated ^15^N labeled pistils were dissected 3 hours after pollination (Fig. 1A), removing both stigma and ovaries, and styles pooled within replicates. Preliminary experiments showed pollen tubes had grown approximately half the length of styles after 3 hours (Fig. 1B). From each ^15^N labeled maternal plant, an equal number of unpollinated flowers were simultaneously collected, dissected, and pooled as negative controls for pollen tube protein identification and to estimate percent ^15^N incorporation (see below).

### Identification of pollen tube proteins via tandem mass spectrometry

Proteins from both pollinated and unpollinated negative control styles were identified using a shotgun proteomic approach utilizing tandem mass spectrometry (MS/MS). Total protein was extracted from pooled pollinated and unpollinated styles for each of the 3 replicate ^15^N labeled plants as in [57], followed by tryptic digestion of the resulting approximately 10 µg of protein as in [15]. Digested proteins (5 µg each) were then analyzed via multi-dimensional protein identification technology (MudPIT) as in [15] using a 13 step (0 to 5 M ammonium acetate) salt elution. In addition, proteins were analyzed by reversed-phase HPLC (5 technical replicates each). For reversed-phase analyses, digested proteins (1 µg per technical replicate) were injected into a 75 µm internal diameter capillary column packed with 30 cm of Jupiter C12 reversed-phase resin, peptides eluted in a 4 hour water∶acetonitrile gradient and mass spectra acquisition handled exactly as for MudPIT analyses.

The acquired tandem mass spectra were searched against a database containing all IM62 protein coding transcripts (JGI release v1.1; http://www.phytozome.org/), proteins of common contaminants (e.g., trypsin, keratin), and a shuffled decoy database using a parallelized implementation of Sequest [76]. Search databases and Sequest search parameters are available for public download (https://sites.google.com/a/uw.edu/maccoss/home/supplementary_data/). The program IDPicker [77], implemented as part of a data analysis pipeline, was used to filter the peptide identifications and assemble peptides and proteins. Protein identification filters (≥1 unique peptide per protein, per peptide q-value≤0.002) were selected to produce protein identifications with an approximate false discovery rate (FDR)≤2% [61]. Pollen tube proteins (PTPs) were then defined as those found uniquely within samples from pollinated,^15^N labeled styles and absent from all negative controls (unpollinated, ^15^N styles). Tandem mass spectra from negative controls were also used to measure the extent of ^15^N labeling of IM62 plants. Equal amounts of digested protein from the 3 replicate negative controls was pooled and analyzed as above for reversed-phase MS/MS, but employing a 3 hour water∶acetonitrile gradient and high resolution mass spectrometry in order to estimate the percent ^15^N incorporation among peptides using the Hardklör algorithm [43].

### Characterization of the *M. guttatus* pollen tube proteome

The constituent proteins of the *M. guttatus* pollen tube proteome were characterized using several quantitative and descriptive measures. The relative abundance of each PTP was inferred using the spectral counting method of [[78]; normalized spectral abundance factor, NSAF] and averaged across MudPIT and reversed-phase analyses from which the protein was identified. Putative functions of PTPs were inferred by automated annotation of all IM62 protein coding transcripts using the Blast2GO annotation tools with default parameters [79]. Fisher's exact test (p≤0.05) was then used for statistical comparison of functional term enrichment [79] between all PTPs and all IM62 coding transcripts, or a subset of PTPs identified in our molecular evolution studies (see below). Similarity among constituent genes of the *M. guttatus* pollen tube proteome, the *Arabidopsis thaliana* pollen proteome [45], and the *A. thaliana* pollen tube transcriptome [46] were inferred from blastp scores [80] from which putative orthologs were identified based on a minimum expectation value of e^−20^.

### *M. guttatus* style proteins

To test for evidence that PTPs may also function in female reproductive structures, we also carried out MS/MS of style proteins. Style proteins were identified and characterized as for PTPs, but using pooled unpollinated styles from a single unlabeled (^14^N) maternal plant. Styles were harvested from virgin flowers within 24 hours of receptivity (gauged by petal opening). Though flowers were not emasculated in the bud, protogyny significantly reduces opportunities for pollen contamination. Style protein digestion, peptide separation, and mass spectra acquisition were carried out as above for MudPIT analyses of PTPs, and acquired tandem mass spectra were searched and assemble into peptides and proteins in an identical fashion as previously (≥1 unique peptide per protein, per peptide q-value≤0.002, approximate protein FDR≤2%). The relative abundance of each style protein was estimated (NSAF).

### Capture and sequencing of pollen tube exomes within a population of *M. guttatus*

Because sexual selection underlying CPP functions as a strong evolutionary force principally at the population level where pollen competition may be high [35], we sequenced PTPs from individuals within a natural population of *M. guttatus* at Cone Peak, Oregon, U.S.A. This population consists of several thousand synchronously flowering and primarily outcrossing annual plants that have been studied previously [e.g., [21], [81], [82]], and is adjacent to the Iron Mountain population from which the IM62 strain used in genome sequencing and genetic mapping studies was derived. A total of 50 individual plants were collected at random on July 11–12, 2011, from locations spanning the population (≥4 m between plants; centroid N 44°24.472′W 122°08.111′, elevation 1,580 m), transported in 50 ml Falcon tubes containing moist potting soil to the University of Washington greenhouses (Seattle, WA), and transplanted into 10 cm pots where they were grown 3 weeks under long day conditions in order to obtain sufficient vegetative tissue for genomic DNA extractions. Individual plant's vegetative tissue for all surviving plants (96% survivorship) were harvested and stored at −80°C.

Coding sequences of PTPs from 28 randomly selected Cone Peak individuals were obtained by array capture and high throughput sequencing. Genomic DNAs were extracted from frozen tissue using the Plant DNeasy Maxi Kit (Qiagen, Valencia, CA) and concentrated using 30K nucleic acid concentration columns (Millipore, Billerica, MA) per manufacturers guidelines. Prior to capture, sequencing libraries were constructed as in [83], except that a unique bar-coded reverse primer was used for each individual in place of the SLXA_Pair_Rev_Amp primer (5′_CAAGCAGAAGACGGCATACGAGATNNNNNNNNCGGTCTCGGCATTCCTGCTGAACCG_3′). This allowed us to pool 14 barcoded individuals for capture and sequencing. A custom Agilent array (244K format; Agilent, Santa Clara, CA) was designed with probes tiling all exons of the 2,554 identified PTPs (Table S1). The probe sequences were pulled from the IM62 reference genome and designed according to [84]. A total of 20 ng of pooled barcoded DNA (1.4 ng per individual) was then captured using the protocol described in [83]. Array-bound DNAs were eluted with two sequential additions of H_2_O (1 ml, 95°C each). Each elution was ethanol precipitated and re-suspended in 20 µl H_2_O, and then PCR amplified as in [83] with forward (5′_AAT GATACGGCGACCACCGAGATCT _3′) and reverse (5′_CAAGCAGAAGACGGCATACGAGAT_3′) PCR primers.

We generated one lane of 76-bp paired-end reads for each of the two pooled sequencing libraries (from the first elution) on an Illumina Genome Analyzer IIx according to the manufacturer's instructions. Sequencing reads from different individuals were divided by their barcode sequence and individually aligned to the IM62 reference genome using BWA v0.5.9 [85] with parameters that were optimized for mapping highly diverged sequences. These modifications included raising the maximum number of differences allowed in the alignment of both the seed region (raised from 2 to 4; -k 4) and the entire read (raised from 4 to 10; -n 10). The alignments were sorted and filtered for duplicates using Picard 1.15 (http://picard.sourceforge.net). GATK [86] was then used to perform local indel realignment and SNP calling across all 28 individuals simultaneously with minimum base and mapping qualities of 20 [87]. SNP calls for each individual were then required to have a minimum genotype quality of 30 and a minimum read depth of 10. For each gene, we calculated standard measures of nucleotide diversity per site (expected heterozygosity) and skews in the site frequency spectra [*Tajima's* D; [64]] after excluding sites with missing data and those that violate the infinite sites model [88].

In order to infer the subset of PTPs that may have been targeted by selective sweeps or balancing selection at Cone Peak, we first utilized the empirical distribution of *Tajima's* D calculated from exome-captured PTPs to establish 95% confidence intervals defining a neutral expectation. Because population demographic forces affect all loci genome-wide, *Tajima's* D values for PTPs in the 2.5% upper and lower tails are likely to reflect locus-specific selective forces assuming loci are unlinked (balancing selection and sweeps, respectively). Such use of an empirical distribution drawn from population genomic data provides a straightforward and robust means of identifying potential targets of positive selection [89]. Next, in order to provide statistical support for these cutoffs we generated 10,000 replicate datasets for each PTP in the upper and lower 2.5% tails of the distribution under a neutral model in the MS program [90] implemented in the DNAsam computer package with default simulation parameters [91]. P-values were calculated for each of these loci as the proportion of 10,000 coalescent simulations with a *Tajima's* D value more extreme than the observed value. Finally, to validate the subset of PTPs identified as potential targets of selective sweeps, we calculated *Fay and Wu's* H [92]. These two statistics use different portions of the frequency spectrum to infer sweeps, with *Fay and Wu's* H relying on high frequency alleles as determined via comparison with an outgroup taxon. While population growth can potentially confound inference of sweeps via *Tajima's* D, *Fay and Wu's* H is generally robust to this aspect of population demographic history. We calculated *Fay and Wu's* H and associated p-values for each PTP in the tails of the empirical distribution as above for *Tajima's* D, utilizing each of two outgroup taxa (*M. tilingii* or *M. cupriphilus*; see below) to infer the ancestral state at variable sites.

### Divergence of PTPs among yellow monkeyflowers

We next tested for evidence of positive selection acting more broadly on PTPs by examining their divergence between Mimulus species. We downloaded publicly available paired-end Illumina sequences from NCBI's Sequence Read Archive (SRA) for 10 accessions of yellow monkeyflowers. These include a dune ecotype of *M. guttatus* along with 8 additional taxa of the *M. guttatus* species complex (*M. cupriphilus*, *M. dentilobus*, *M. glaucescens*, *M. micranthus*, *M. nasutus*, *M. nudatus*, *M. pardalis*, and *M. platycalyx*), and a closely related outgroup, *M. tilingii* [SRX030540-1, SRX030973-4, SRX116529, and SRX142372-6; [17], [18]]. Read mapping and variant calling were preformed in the same way as described for the Cone Peak individuals. Coding sequences were extracted from the genotype calls and required to contain a minimum of 75% unambiguous bases per gene.

We examined the evolutionary forces acting among PTPs and other CDSs genome wide by comparing the rate of non-synonymous (*d*_N_) with synonymous (*d*_S_) nucleotide substitutions. The ratio (*d*_N_/*d*_S_, or ω) constitutes an index of selection where ω<1 is consistent with purifying selection, ω = 1 indicates neutral evolution, and ω>1 is consistent with positive selection (i.e., adaptive diversification). We first calculated pairwise estimates of *d*_N_ and *d*_S_ for each CDS between IM62 and the outgroup *M. tilingii* to examine their respective distributions across the species complex using codeml in the PAML computer package [93]. We then estimated *d*_N_/*d*_S_ directly from multiple sequence alignments containing a minimum of 3 taxa with the one-ratio sites model in codeml [M0; [94]]. Finally, we explicitly tested for positive selection acting on individual PTP CDSs using nested sites models that either allow for positive selection at a subset of codons (M8) or constrain ω≤1 (M8a). Neighbor joining (NJ) trees were constructed for each CDS to avoid potential confounding effects from introgression among species. We assessed statistical significance by performing likelihood ratio tests with a χ^2^ approximation to calculate P-values.

## Supporting Information

Figure S1 Comparison of pollen tube proteins (PTPs) and style proteins identified among tandem mass spectrometry (MS/MS) experiments. (A) PTPs from styles of three independently ^15^N labeled plants (red, blue, and green circles) pollinated with unlabeled (^14^N pollen) and identified using reverse phase and MuDPIT chromatography in combination with MS/MS. Results from reverse phase and MuDPIT experiments were pooled within plants (full replicates), and the total number of PTPs identified from each replicate are indicated. Overlap among replicates is substantial, with ∼60% of PTPs (2,554 in total) shared between any two experiments and identification of 36% common among all three replicates. (B) PTPs identified by either reverse phase or MuDPIT chromatography (grey or black circles, respectively) in combination with MS/MS. Results from replicate plants were pooled and the total number of PTPs identified using either method of peptide separation are indicated. MuDPIT identified nearly twice the number of PTPs as reverse phase chromatography, with nearly complete overlap in the PTPs identified (97%). (C) Overlap between cumulatively identified PTPs (yellow circle) and style proteins identified by MuDPIT in combination with MS/MS from unlabeled plants (^14^N styles; green circle). Approximately half of protein identifications are shared between pollen tubes and styles.
(TIF)

File S1 Constituent proteins of the pollen tube and style proteomes of yellow monkeyflowers. **Pollen Tube Proteins Worksheet.** The inbred strain of *Mimulus guttatus* (IM62) used for genome sequence construction was isotopically labeled with ^15^N via hydroponic culture and used as the pistil (maternal) parent in crosses to unlabeled (^14^N) IM62 pollen parents. Because ^15^N labeling masks maternal proteins in our MS/MS shotgun proteomic method (see results), proteins can be identified as originating from *in vivo* pollen tubes unambiguously. Pollen tube proteins (PTPs) are identified from tandem mass spectra (MS/MS) matched to proteins from gene model numbers corresponding to the *M. guttatus* genome assembly version 1.1 (http://www.phytozome.net) using SEQUEST [76], and require ≥1 unique peptide per protein with a low false discovery rate (per peptide FDR≤0.002). Proteins are identified as unique (2,073) or members of a multi-protein group (481) which could include 1 or more member proteins [95]. Protein group numbers are arbitrarily assigned, and unless protein identifications are unique (*), the multiple proteins within a group cannot be further distinguished in the data. The number and sequence of peptides identified for each PTP are indicated (summed over all experiments), along with a measurement of the protein's relative abundance (normalized spectral abundance factor, NSAF) [78] in each of three biological replicates analyzed via MS/MS utilizing either 1-dimensional (reverse phase) or multi-dimensional (MuDPIT [96]) chromatography for separation of peptides. **Style Proteins Worksheet.** Proteins from styles of unpollinated pistils of unlabeled (^14^N) IM62 plants were identified as for PTPs employing MuDPIT in combination with MS/MS, yielding 1988 unique style proteins (*) as well as 620 proteins that are members of multi-protein groups. Approximately 50% of the proteins identified from style tissue were also found in pollen tubes (Fig. S1).
(XLS)

File S2 Relative enrichment of gene ontology terms among pollen tube proteins. Automated annotation [79] of the *Mimulus guttatus* genome assembly version 1.1 (http://www.phytozome.net) which contains 28,274 coding sequences yielded 61% with annotations genome wide. Of 2,554 PTPs identified (Table S1), 91% are in the genome annotation set. Comparison of the number of PTPs relative to all other coding sequences assigned to a given GO term classification across multiple levels identified many GO term classifications that were significantly over or under represented among PTPs as determined from Fisher's exact test (FDR = 0.05) [79].
(XLS)

File S3 Molecular Evolution of *Mimulus* Pollen Tube Proteins. **Adaptive Divergence Worksheet.** For the 2,554 pollen tube proteins (PTPs) identified, we assembled and mapped publicly available (NCBI SRA) Illumina reads for 10 members of the *M. guttatus* species complex (*M. tilingii*, *M. platycalyx*, *M. pardalis*, *M. nudatus*, *M. nasutus*, *M. micranthus*, *M. glaucescens*, *M. dentilobus*, *M. cupriphilus*) and a dune ecotype of *M. guttatus* [17] to the *M. guttatus* (IM62 v. 1.1) reference sequence (phytozome.org). Sequences were assembled into alignments for each PTP if they covered >75% of the IM62 reference CDS, from which codon substitution models (M8a and M8) were fit using codeml in the PAML computer package [93] with CDS-specific neighbor joining topologies. The p-values from χ^2^ -tests comparing models M8a with M8 are shown for each PTP, which constitute statistical support for the model (M8) that identifies a subset of sites as being under positive selection; corresponding q-values [97] control for multiple testing, with significance among PTPs set at a false discovery rate (FDR) of 0.10. Manual validation of the subset of alignments with FDR<0.10 confirmed statistical support for positive selection for all but two PTPs (*). **Polymorphism and Selection Worksheet.** PTP Exomes of 28 wild collected individuals from the Cone Peak population of *M. guttatus* [81] were captured on a custom Agilent micro-array and Illumina sequenced, after which reads were assembled and mapped to IM62 CDSs. Mean read depth was high (133/bp), with 92% of the target sequence for the 2,554 PTPs covered at a minimum read depth of 10. Base calls were made for sites with ≥10 reads, from which measures of nucleotide diversity per site (expected heterozygosity) and *Tajima's* D [64] were made after excluding sites with missing data and those that violate the infinite sites model [88]. The empirical distribution of *Tajima's* D among 2,352 PTPs shows no evidence of confounding demographic forces at Cone Peak (see Fig. 3B), thus the upper and lower tails of the distribution (2.5% each, in bold) were established as a 95% confidence interval beyond which genes may be targets of sweeps or balancing selection (negative or positive D, respectively). Coalescent simulations under a neutral model for each of these 116 genes (10,000 per PTP) implemented in the DNAsam computer package under default simulation parameters reflecting a neutral coalescent model [91] provide p-values that validate the significance threshold established from the empirical distribution (i.e.,p≤0.025). For the 58 genes identified as potential targets of sweeps, Fay and Wu's *H* statistic [92] was calculated in DNAsam using orthologs from each of two separate outgroups (*M. tilingii* or *M. cupriphilus*). P-values calculated via coalescent simulations as above validate results from *Tajima's D* for 44 of these PTPs (76%).
(XLS)
