# Supplementary figures and images for: Selection on Plant Male Function Genes Identifies Candidates for Reproductive Isolation of Yellow Monkeyflowers
Source: PLoS Genet. 2013 Dec 5;9(12):e1003965. doi: 10.1371/journal.pgen.1003965 (PMC3854799; doi:10.1371/journal.pgen.1003965)

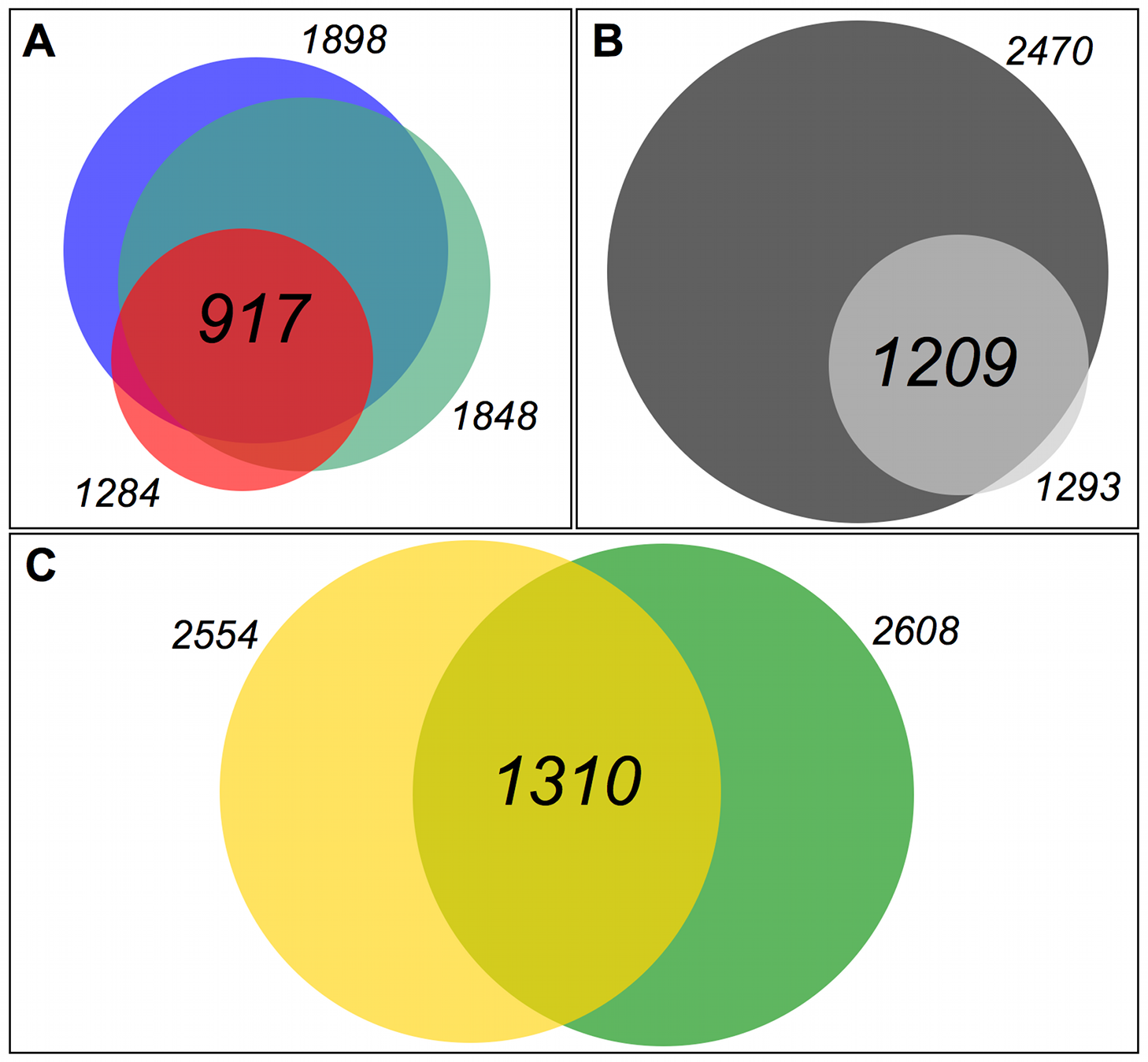

Supplement: Figure S1 — Comparison of pollen tube proteins (PTPs) and style proteins identified among tandem mass spectrometry (MS/MS) experiments. (A) PTPs from styles of three independently 15N labeled plants (red, blue, and green circles) pollinated with unlabeled (14N pollen) and identified using reverse phase and MuDPIT chromatography in combination with MS/MS. Results from reverse phase and MuDPIT experiments were pooled within plants (full replicates), and the total number of PTPs identified from each replicate are indicated. Overlap among replicates is substantial, with ∼60% of PTPs (2,554 in total) shared between any two experiments and identification of 36% common among all three replicates. (B) PTPs identified by either reverse phase or MuDPIT chromatography (grey or black circles, respectively) in combination with MS/MS. Results from replicate plants were pooled and the total number of PTPs identified using either method of peptide separation are indicated. MuDPIT identified nearly twice the number of PTPs as reverse phase chromatography, with nearly complete overlap in the PTPs identified (97%). (C) Overlap between cumulatively identified PTPs (yellow circle) and style proteins identified by MuDPIT in combination with MS/MS from unlabeled plants (14N styles; green circle). Approximately half of protein identifications are shared between pollen tubes and styles. (TIF) [file pgen.1003965.s001.tif]
